# When Attention Mistakenly Becomes Authority

**DOI:** 10.1111/jocd.71192

**Published:** 2026-10-12

**Authors:** Steven Dayan

**Affiliations:** ^1^ University of Illinois Champaign Illinois USA

Medicine has always depended on trust. Long before clinical trials, practice guidelines, and evidence‐based medicine became the foundation of modern healthcare, patients entrusted medical professionals with difficult decisions because they believed in their expertise earned through years of study, observation, experience, and thoughtful judgment. Authority was never intended to be a popularity contest. It was earned slowly, questioned continuously, and granted reluctantly. Today, however, we increasingly confuse attention with authority, where followers, views, and engagement often speak louder than scholarship and experience.

The modern educational pathway in aesthetic medicine is unlike almost every other medical specialty. There is no universally accepted certifying body that defines true expertise for an injector or aesthetic provider. For example, I am board certified to do cervical pontine brain stem surgery, but despite my certification, you don't want me to do it because I haven't done it since residency. But you do want me to do your rhinoplasty, facelift, or filler injection, something I have expertise in, do every day, and have contributed extensively to the literature.

Into the void of defining expertise has stepped the opportunistic social media personalities. Instagram, TikTok, and YouTube, which have now become the classroom and showroom for an entire generation. Everyday thousands of less than discerning consumers and practitioners absorb nuggets of information, watch techniques and gape at polished outcomes from reels, videos and posts designed to stop a scrolling thumb. The incentive for the creator is not to be correct but to peacock their expertise and be captivating.

Captivation and education, however, are not the same thing. My applause goes out to Cotofana and colleagues who recently published an international analysis of more than 900 aesthetic healthcare professionals across the globe and asked a surprisingly simple question: does social media popularity correlate with academic contribution? Their answer was equally straightforward [1]. Among the professionals studied, nearly three quarters of Instagram personalities had never authored a single indexed publication despite averaging almost 57 000 followers, while nearly two thirds of TikTok personalities likewise had no publications despite averaging almost 80 000 followers. Most striking was the absence of any meaningful relationship between follower count and scientific output. Popularity and scholarship appeared to be measuring entirely different things.

That finding should give every educator, conference organizer, industry executive, journalist, and young physician pause. If attention is increasingly directed toward those who entertain rather than those who investigate, we should not be surprised when evidence and good medicine give way to opinion and misguided collective wisdom.

This is not an indictment of social media. Social media has democratized education in remarkable ways, and I have learned valuable pearls from clinicians I might never have gotten otherwise. Brilliant educators have reached audiences that traditional academic channels could never have touched. The problem is not the platform; rather, it is those who confuse visibility with expertise.

Medicine has always required a healthy dose of skepticism and uncertainty. Experienced physicians rarely speak in absolutes because they have learned that biology has a way of humbling. Treatments evolve, preferred techniques change, and long‐held beliefs are overturned. The physician who has practiced for decades usually becomes more careful with language, not less. Social media, by contrast, often rewards cocky assuredness, flashy novelty, and provocative claims that capture attention in under 3 s.

Every week in my own practice I meet patients seeking second opinions after consulting physicians who have become enormously popular online. Many are talented clinicians and gifted communicators. Others, however, have accumulated extraordinary influence before accumulating extraordinary experience. That is not a criticism of youth because every experienced physician was once an inexperienced physician. Throughout my career I have had the privilege of mentoring hundreds of residents, fellows, and young practitioners. Innovation often begins with younger physicians who ask questions a previous generation stopped asking years ago. What concerns me is not youth but certainty without scrutiny and posturing without humility. I see disproportionate praise for the flash in my feeds, on the pages of trade journals and the stages at conferences.

One of the quiet truths of publishing is that it changes the author as much as it changes the literature. Over the course of my career I have participated in more than 225 peer reviewed publications and numerous clinical trials. Many early ideas were deservedly rejected. Others required better study design, stronger statistics, or simply clearer thinking. Peer review can be frustrating, but it is also profoundly educational because it forces us to ask whether our observations are reproducible, whether our conclusions are justified, or if alternative explanations deserve consideration. Those questions make us rethink and refine our ideas. We become more logical in our thinking, more humbled in our convictions, and we are forced to become better clinicians, long before being a recognized expert or published author.

Publishing is certainly not a perfect measure of expertise, nor does authorship automatically confer wisdom or technical excellence. Outstanding clinicians may never write a paper. At the same time, contributing to scientific literature demonstrates something increasingly valuable. It reflects a willingness to have one's ideas challenged before asking colleagues or patients to believe them.

Perhaps the solution is not to choose between scholars and influencers. Perhaps we should expect and attempt to blend both. The physician capable of translating complex science responsibly to a broad audience may ultimately become the educator our 21st century specialty needs. Industry can help by rewarding thoughtful published educators rather than simply those with the largest or right audiences. Conference organizers can grant more podium time to good communicators with vetted ideas; journalists can cross‐reference those they choose to quote based on academic excellence and responsible posting and medical journals can create more accessible pathways for novice clinicians with important observations to publish.

Too often I hear physicians say they are too busy to publish, and I understand that sentiment. Clinical practice is demanding, research receives little direct financial reward, and rejection can be discouraging. Yet every meaningful contribution to medicine began with someone deciding that an observation deserved more than a social media post. If you have an idea worth teaching, it is worth testing and publishing. If you need help or are interested in learning how to conduct trials or publish, ask someone whose work you admire. Most experienced investigators are remarkably generous because someone once did the same for them.

Artificial intelligence may ultimately shift this conversation again. Increasingly, large language models give greater weight to peer‐reviewed literature and established academic sources when synthesizing medical information. Clicks, followers, likes, and shares may become less important in the coming years as AI seems to prefer unique and authentic contribution to the scientific record. I welcome this advancement because medicine has never been about collecting followers. It has always been about putting the patient's best interest first. Long after the social media algorithm has forgotten our posts, the ideas we contributed to medicine will continue for the next generation of professionals and patients whom we will never meet.

The greatest legacy of a medical professional has never been the audience we attracted, but the people whose lives we improved, and the knowledge left behind for others to build upon.

## Conflicts of Interest

The author declares no conflicts of interest.

## Data Availability

Data sharing not applicable to this article as no datasets were generated or analyzed during the current study.
